# Growth Hormone Therapy Benefits Pituitary Stalk Interruption Syndrome Patients with Short Stature: A Retrospective Study of 75 Han Chinese

**DOI:** 10.1155/2016/1896285

**Published:** 2016-04-13

**Authors:** Cheng-Zhi Wang, Ling-Ling Guo, Bai-Yu Han, An-Ping Wang, Hong-Yan Liu, Xing Su, Qing-Hua Guo, Yi-Ming Mu

**Affiliations:** ^1^Department of Endocrinology, Chinese PLA General Hospital, Beijing 100853, China; ^2^Department of Endocrinology, Beijing Electric Teaching Hospital of Capital Medical University, Beijing 100073, China; ^3^Department of Endocrinology, Hainan Branch of Chinese PLA General Hospital, Sanya, Hainan 572000, China

## Abstract

*Objective*. We aim to investigate the long-term benefits of growth hormone (GH) therapy in short stature adolescents and adults with pituitary stalk interruption syndrome (PSIS), which would be beneficial for future clinical applications.* Design and Methods*. In this study, initial height, final height, total height gain, and GH treatment history were retrospectively investigated in 75 Chinese PSIS patients. We compared height gain between the GH treated cohort and untreated cohort and explored the impact of different GH therapy duration on height gain.* Results*. For GH treated patients, their final height (SDS) increased from −1.99 ± 1.91 (−6.93~2.80) at bone age (BA) of 11.2 (5.0~17.0) years to −1.47 ± 1.64 (−7.82~1.05) at BA of 16.6 (8.0~18.0) years (*P* = 0.016). And GH treated patients had more height gain than the untreated patients (*P* < 0.05). There was a significant difference between the different GH therapy duration groups (*P* = 0.001): GH 0 versus GH 3, *P* = 0.000; GH 1 versus GH 3, *P* = 0.028; GH 2 versus GH 3, *P* = 0.044.* Conclusion*. Adult Chinese PSIS patients with short stature benefited the most from at least 12 months of GH therapy. Although patient diagnosis age was lagged behind in the developing countries, GH treatment was still effective for them and resulted in a higher final height and more height gain.

## 1. Introduction

Pituitary stalk interruption syndrome (PSIS) is a rare congenital defect that manifests with varying degrees of anterior pituitary hormone deficiency [1–5]. Because its diagnosis requires MR imaging (MRI), many cases are initially diagnosed as growth hormone deficiency (GHD), isolated hypogonadotropic hypogonadism (IHH), or other types of hypopituitarism because of the similar clinical manifestations. With the development of imaging technologies, PSIS was gradually discovered and found to cause a small number of hypopituitarism cases.

Currently, PSIS cannot be predicted before birth, and its causes are still elusive. Genetic findings and high frequency of prenatal events cannot fully explain the disease pathogenesis [2–4, 6–8]. Once diagnosed, a PSIS patient, he (she), may need lifetime hormone replacement therapy and is likely infertile. Because patient quality of life is greatly influenced by the disease, critical evaluations of the current treatment benefits are of importance.

Very few studies of PSIS treatments have been done, and evaluation of GH therapy is limited to early treatment in children [9–11]. In these studies, catch-up growth was estimated in the early years of the therapy [11–13]. Whether patients reached a satisfactory final height in adulthood remains unclear. The impact of GH treatment on final height and total height gain compared to untreated patients is also unknown. In our study, we retrospectively reviewed 75 PSIS patients with short stature in our hospital over the last 10 years. We revisited the patients and analyzed their treatment records and measured their final height, height gain, and hormonal status. This novel information will be instructive for PSIS treatment in future clinical practices.

## 2. Subjects and Methods

### 2.1. Participants

From 2004 to 2014, we collected information from 75 PSIS patients with short stature (66 male, 9 female) who had revisited our hospital within the past two years. These patients were diagnosed out of the 251 hospitalized hypopituitarism patients in our hospital. They presented with the typical PSIS manifestations of a thin pituitary gland, ectopic neurohypophysis, and an interrupted stalk on MRI. We defined short stature as a height of more than two standard deviation scores (SDS) below the mean for the patients' chronological age (CA).

Clinical features and medical histories of the patients were retrospectively reviewed. Data were collected by telephone calls with patients, from previous medical records of local hospitals and from the patient record system of our center. Medical record information included the following: consanguineous parents, family medical history, perinatal history (gestational age, delivery, and neonatal hypoxemia), medication history (including prednisone), hormone treatments (thyroid hormone, sex hormone, or gonadotropins), associated malformations (microphallus, cryptorchidism, or midline abnormalities), other diseases, pubertal status, chronological age, bone age, and initial height.

This study was approved by the Ethics Committee of the Chinese PLA General Hospital. Informed consent from all participants was obtained prior to their participation in this study.

### 2.2. Treatment

For GH therapy, recombinant human growth hormone was given by subcutaneous injection for six days a week to patients who were willing to grow taller with unclosed osteoepiphysis. The total dose was 0.10–0.2 iu/kg per day, and the GH therapy duration was evaluated based on height gains. GH treatment occurred prior to PSIS diagnosis in some patients because they had been treated for GHD elsewhere.

### 2.3. Auxological Measurements

In our study, height is expressed in SDS according to the actual bone age (BA) measured by X-ray film for the patients' BA was delayed to CA. Short stature was defined as a height more than 2 SDS below the mean for the patient's CA. For patients receiving GH treatment, the total height gain was defined as the difference between the patient's final height at the last visit and their initial height at the first visit. For untreated patients, height difference was defined as the patient's final height at the last visit minus their initial height when they were diagnosed with GHD and refused treatment at the local hospital.

### 2.4. Hormonal Evaluations

GHD was confirmed by pyridostigmine bromide test and insulin-induced hypoglycemia tolerance test (ITT). Peak GH values of less than 5 ng/mL were diagnosed as complete GH deficiency (CGHD) and 10 ng/mL as partial GH deficiency (PGHD). Pituitary hypothyroidism was diagnosed if basal serum free T4 (FT4) levels were below normal (<10.42 pmol/L). ACTH deficiency was diagnosed when morning basal serum cortisol levels were less than 198.7 nmol/L with no significant increase during hypoglycemia. LH/FSH deficiency was diagnosed based on delayed or absent pubertal development, low serum levels of testosterone for males (<8.4 nmol/L) or estradiol for females (<48.2 pmol/L), and blunted LH/FSH response to a GnRH stimulation test.

### 2.5. Statistical Analyses

All data are presented as mean ± SD/(extreme values), medians (extreme values), or percentages. The Mann-Whitney *U* test, Kruskal-Wallis test, *t*-test, and Wilcoxon test were used to compare means between groups and the *χ* ^2^ test was used for percentage analyses. The relationships between variables were tested by Spearman correlation coefficient. The threshold of statistical significance was 0.05, unless otherwise mentioned. Statistical tests were performed using the SPSS 21.0 statistical software package (SPSS, Chicago, IL, USA).

## 3. Results

### 3.1. Initial Patient Characteristics

The ratio of males to females was 7.3 : 1 for the 75 participants (66 male, 9 female). Patients were diagnosed with PSIS at 21.25 ± 6.21 (7.0~43.0) years, and their average current CA was 25.53 ± 6.24 (10.0~48.0) years. In the GH treated cohort, treatment was initiated at 18.24 ± 5.11 (9.0~35.0) years. Patients in the untreated cohort were diagnosed with GHD at 18.96 ± 6.58 (7.0~33.0) years and refused to use GH that was suggested by their doctor. Sex chromosome findings matched the gender phenotypes. There were no documented consanguineous parents for any of the patients. 68 of the patients were breech or footling deliveries (68/75, 90.7%), and 41 patients experienced perinatal events including dystocia or suffocation (41/75, 54.6%). Only five patients had midline abnormalities. One had a partial absence of the corpus callosum, and the others suffered from Chiari-I malformation and syringomyelia. All cases had GHD. General characteristics of the GH treated and untreated groups are summarized in Table 1.

### 3.2. Final Height Changes after GH Treatment

For all 75 cases, though no statistical significance was shown, the average patient final height (SDS) increased from −2.10 ± 1.76 (−6.93~2.80) to −1.86 ± 2.04 (−7.82~4.12). The average height gain for all patients was 0.19 (−4.55~6.46).

Fifty-one patients received recombinant GH treatment. Patients started their treatment at 18.24 ± 5.11 (9.0~35.0) years of CA with a BA of 12.1 (5.0~17.0) years, and their current CA was 24.78 ± 5.22 (12.0~41.0) years with a corresponding BA of 16.6 (8.0~18.0) years. The average therapy duration was 10.0 (3.0~60.0) months. All the treated patients, except two boys still on the treatment at the age of 12 and 10 years, had completed their GH therapy. Their final heights were higher than the heights before treatment: −1.99 ± 1.91 (−6.93~2.80) at BA of 11.2 (5.0~17.0) years versus −1.47 ± 1.64 (−7.82~1.05) at BA of 16.6 (8.0~18.0) years, *P* = 0.016.

**Figure 1:**
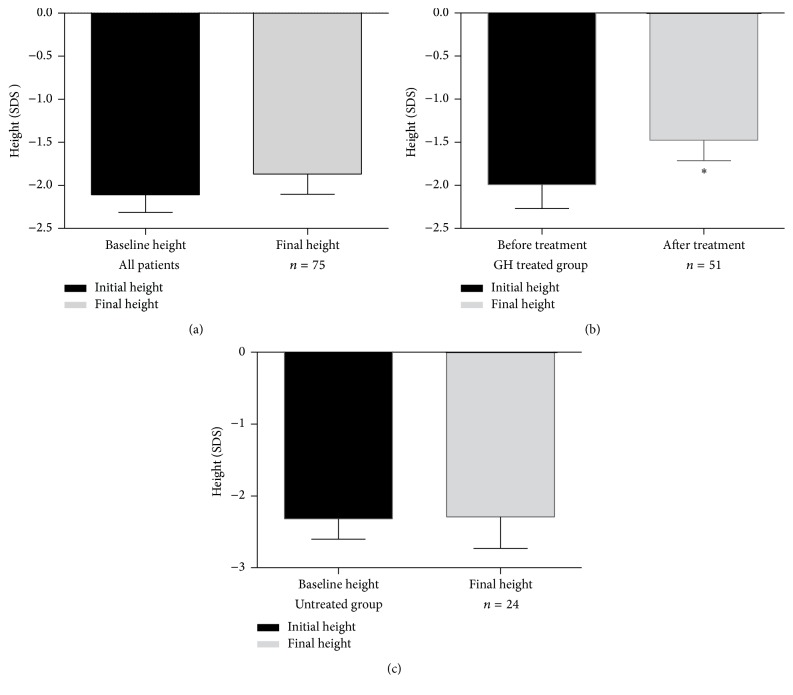
Final height changes after GH treatment. (a) Height changes for all the participants. (b) Height changes for GH treated cohort. (c) Height changes for untreated cohort ^*∗*^ *P* < 0.05.

In the other 24 patients who did not use GH, their average height (SDS) changed from −2.31 ± 1.48 (−6.18~0.50) at BA 11.8 (5.0~17.0) to −2.56 ± 2.50 (−6.18~4.12) at BA 16.9 (8.0~18.0) without statistically significant difference. Above trends were presented in Figure 1.

### 3.3. GH Treatment Increased the Average Height Gain and Final Height in Patients

The average height gain (SDS) was 0.55 (−4.55~6.46) for GH treated patients (*n* = 51) and 0.34 (−4.53~5.90) for untreated patients (*n* = 24). The height increase in GH treated patients was significantly more than in untreated patients (*P* = 0.011).

Final height in GH treated group was also higher than the untreated ones, −1.47 ± 1.64 (−7.82~1.05) versus −2.56 ± 2.49 (−6.18~4.12) (*P* < 0.05).

Notably, the final heights of three males and one female patient in the untreated cohort were increased to 1.75 ± 1.49 SDS without any GH supplementation, even though they were diagnosed with short stature and GHD during childhood.

### 3.4. Treatment Duration and Total Height Gain

For the 51 patients who received GH therapy, the average treatment duration was 10.0 (3.0~60.0) months. 15 patients received GH injections for less than 6 months (GH 1 group), 13 patients for 6 to 12 months (GH 2 group), and 22 patients for more than a year (GH 3 group). We further compared height gains for these three groups and the untreated group (GH 0 group) and found that there was a significant difference between the four groups (*P* = 0.001).

**Figure 2:**
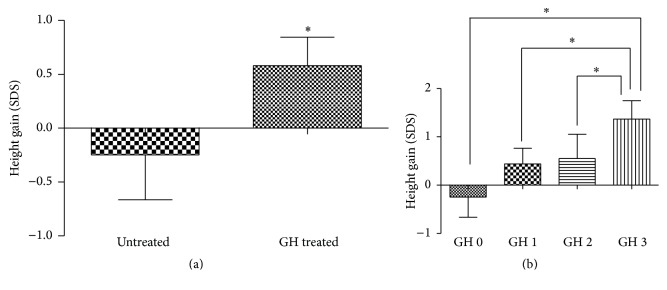
Comparison of height gain. (a) Height gain between the GH treated cohort and untreated cohort. (b) Height gain among different GH therapy duration ^*∗*^ *P* < 0.05.

Statistically significant differences were found between the following groups: GH 0 and GH 3 −0.24 (−4.53~5.90) versus 1.36 (−2.32~6.46), *P* = 0.000, GH 1 and GH 3 −0.44 (−1.13~3.22) versus 1.36 (−2.32~6.46), *P* = 0.028, and GH 2 and GH 3 −0.30 (−4.55~4.61) versus 1.36 (−2.32~6.46), *P* = 0.044.  Figure 2 had shown the comparisons of height gain.

### 3.5. Correlation between BA before Treatment and Delayed BA and Height

**Figure 3:**
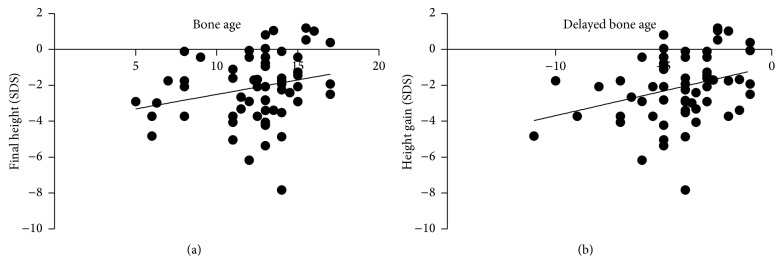
Correlation between bone age/delayed bone age and final height. (a) Correlation between bone age and final height. (b) Correlation between delayed bone age and height gain.

There was a slight positive correlation between initial BA and final height for all 75 patients (*r* = 0.255, *P* = 0.039). Delayed BA was correlated with height gain (*r* = 0.263, *P* = 0.028). The variables are displayed in a scatter diagram (Figure 3).

### 3.6. Hormone Deficiencies and Other Hormone Replacement during GH Treatment

We compared final height and height gain in the following patient groups divided according to hormone status: TSH deficiency or not; ACTH deficiency or not; LH/FSH deficiency or not; pan anterior pituitary hormone deficiency or not. None of these comparisons were significantly different.

Other hormone replacements, including prednisone, thyroid hormone, sex hormone, and gonadotropins, used during GH treatment were also recorded in our study. In the GH treated cohort (*n* = 51), final heights were significantly higher in patients who used sex hormone or gonadotropins (*n* = 20) (−0.87 ± 0.96 versus −1.97 ± 1.90, *P* = 0.025). In the untreated patients (*n* = 24), 14 who used sex hormone or gonadotropins had significantly higher final heights than the 10 patients who had not used the hormones (−1.09 ± 2.36 versus −3.45 ± 1.97, *P* = 0.012), as well as more height gain (−0.80 ± 2.56 versus −0.88 ± 1.43, *P* = 0.039).

**Figure 4:**
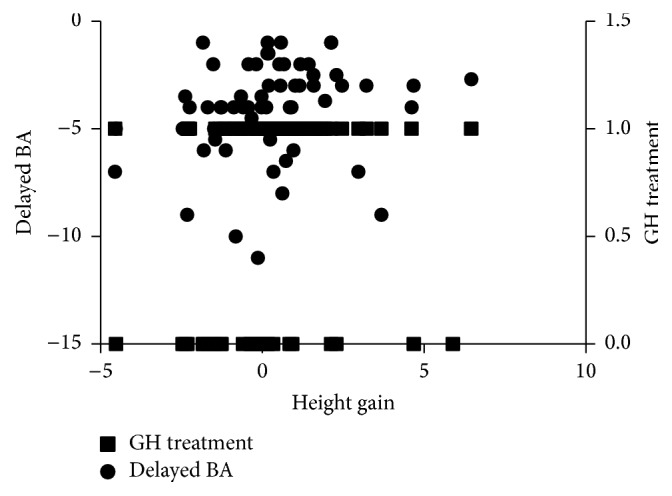
Regression analyses for height gain. horizontal axis: height gain; left vertical axis: delayed bone age; right vertical axis: GH treatment (yes for 1 and no for 0).

Multiple regression analyses were performed. Height gain was set as the dependent variable (*y*), and the factors listed above were independent variables. Only two factors fit the model: GH treatment (have or not) (*a*) and delayed BA (*b*). Model equation was *y* = 0.615 + 1.319*a* + 0.230*b* (*R* ^2^ = 0.375, *P* = 0.006). The model is displayed in Figure 4.

## 4. Discussion

PSIS is a rare congenital defect whose cause is still unclear [14, 15]. Until recently, this disorder could not be predicted before birth, and its diagnosis relied on MRI findings.

Compared to previous research, patients in our study were diagnosed with PSIS at a much older age [9–11, 16–18]. The average age of the 75 subjects we followed was 18.47 ± 5.74 years before treatment. Two patients (one male and one female), however, made their first visit to a local general hospital for short stature in their late 30's. Several reasons can explain the older average age of our patients. First, China has many lower cultural and economic levels. In fact, most cases admitted to our center were from undeveloped areas. Chinese parents are unlikely to send their children to the hospital for systematic inspection until the children display short stature or absent/arrested puberty, regardless of their perinatal events at birth. Additionally, GH treatments are very expensive and are not covered by medical insurance currently, causing some patients to refuse this therapy. Another reason, illustrated in Table 1, is that at baseline the untreated patients had an older BA compared to the GH treated patients. These data suggest that patients may have endured their short stature and had slightly height gain by themselves in the following years. The delayed diagnoses and older BA at the initial visits of the untreated patients caused a slight imbalance in our data. A larger sample size would be beneficial to achieve more explicit results and balance the data.

Unfortunately, since most participants were young adolescents or adults, their growth rates were not recorded every year. Also, their height immediately after treatment was not taken, for they were not routinely followed up like children. However, these patients cared mostly for how much they could grow by using GH therapy and achieving a satisfactory final adult height. In our study, of all 75 patients initially diagnosed with short stature, 34 reached a height of −0.85 (−1.92, 3.98) for their CA and were no longer considered short. We have noticed that the proportion was 41.2% (21/51) in the treated cohort and 51.3% (13/24) in the untreated cohort with no statistically significant differences. As the number of patients is limited in the untreated group (*n* = 24), a larger sample size would be beneficial to achieve more explicit results.

In our cohort, patients began GH treatments at 14.52 ± 5.26 (7.0~30.0) years old, and the average therapy duration was 14.55 ± 11.88 months. Remarkably, despite the delay in diagnosis age and start of treatment, GH therapy had an effect on the total height gained in our study. The height gained by GH treated patients was significantly more than the untreated patients. For example, one male started GH treatment at the age of 25 years at a height of 150 cm. His BA was just 8 years when he was 22. He used GH for one year, and his height increased to 162 cm by 33 years old. When analyzing GH therapy duration and height gain, we found statistically significant differences between the following groups: GH 0 versus GH 3, GH 1 versus GH 3, and GH 2 versus GH 3. These differences suggest that GH treatment for longer than 12 months caused more height gain. This treatment time period is in accordance with China's physician recommendations for GHD patients. Although studies have shown that the best results are achieved with early GH treatment [9, 12, 13, 19], our results indicate that beginning GH treatment later can still be beneficial to adolescent and adult patients. These results have important implications for developing countries where there are significant barriers for PSIS patients.

Interestingly, we found untreated patients who achieved height gain without exogenous GH. Three untreated males reached a normal final height without medical intervention (1.75 ± 1.49 SDS). In their 20's, these patients all had lagging catch-up growth similar to a constitutional delay of growth. One male even grew to 197 cm (3.98 SDS) at the age of 31 years with a BA of 16 years. This patient recalled that his height accelerated after the age of 22 years at a rate of 6 cm per year. Though osteoepiphysis was unclosed, average patient BA at baseline (16.5 years old) was higher than other studies of GH treated patients. Accordingly, bone age/delayed bone age should be measured and used to evaluate which patients need to be treated upon their first visit to the hospital. And the relationship between BA/delayed BA and final height/height gain seemed to give us a clue that BA/delayed BA may be a predictor for final height and thus it should be followed up closely once after a patient had been diagnosed. There may be other compromising factors that contributed to PSIS patients' height even when GHD had been determined. When bone epiphysis was not completely closed, partly because of lack of sex hormones, the extremely low levels of GH and other hormones like insulin, thyroid hormone, and sex hormone could have still contributed to growth. This idea is further supported by our data that patients who had used sex hormone or gonadotropins in both the GH treated and untreated cohorts had higher final height/height gain than patients who had not used the hormones. It is commonly known that sex hormones like estrogen and androgen can stimulate growth and promote epiphyseal fusion [20–22]. All the above beg the question of whether we should “stop” growth in these very tall patients and when and how to “stop” it since their growth curves are not similar to that of children. It is known that IGF1 is an excellent index for evaluating growth; in our study, not enough IGF1 (before and during GH therapy) data were collected and we suggest that this should be routinely measured at the onset and during the GH treatment in future works, which would undoubtedly help the clinical doctors on the adjustment of GH dose and time to finish the course.

In PSIS patients with additional hormone deficiencies, we found lower initial heights than other patients with no additional deficiencies. Combined hormone deficiencies may cause more severe symptoms like short stature [15], and thus patients were more willing to receive GH therapy. Patients who had used sex hormone or gonadotropins during GH therapy had higher final heights than those who had not.

In our study, we noted that 20 patients had fatty liver or dyslipidemia. Three patients had hyperuricemia, and four had osteoporosis. These symptoms may be attributed to the hypothyroidism caused by PSIS and the side effects of prednisone. Side effects of adult GH treatment are reported in up to 30% of patients and are usually dose-dependent. Adverse events include myalgia, carpal tunnel syndrome, edema, elevated blood pressure, left ventricular remodeling, hyperglycemia, and clinical diabetes [23]. Previous studies have shown no evidence that GH therapy causes death, cancer, diabetes mellitus, cardiovascular events, or intracranial tumor growth and recurrence [24].

Even though PSIS is rare, patients suffering from it have the right to have normal lives. Although the age at diagnosis is greater in developing countries because of the economy and health care levels, GH treatment was still necessary and effective. People of short stature with PSIS benefited from GH therapy, and hormone replacement treatments improved patient mental health and the quality of their lives. As hormonal deficiencies worsen in PSIS patients, hormonal evaluations should be performed regularly [25, 26]. Currently, more attention is being given to rural health care in China. Early screenings for newborns have been popularized and the numbers of grant applications for rare diseases as varieties of hypopituitarism have increased. These new developments are promising that PSIS patients in China will receive better treatment and follow-up care.

## Figures and Tables

**Table 1:** Initial characteristics of the 75 patients.

	GH treated cohort (*n* = 51)	Untreated cohort (*n* = 24)
Sex (male/female)	45/6	21/3
Chronological age before treatment (year)	18.24 ± 5.11	18.96 ± 6.58
Bone age (year)	12.1 (5.0~17.0)	13.0 (9.0~17.0)^*∗*^
Delayed bone age (year)	−4.0 (−11.0~−1.0)	−4.0 (−6.0~−1.0)
Height (SDS)	−2.08 (−6.93~−2.80)	−2.25 (−6.18~−50)
Familial history	0	0
Malformations	4	1
Breech presentation (%)	93.3	83.0
Perinatal events (%)	51.1	53.3
GHD (%)	100	100
GHD + ACTH deficiency (%)	62.2	56.6
GHD + TSH deficiency (%)	68.9	70.0
GHD + LH/FSH deficiency (%)	95.6	86.6

^*∗*^ *P* < 0.05, untreated cohort compared to GH treated cohort.

## Abbreviations

PSIS: Pituitary stalk interruption syndrome
IHH: Isolated hypogonadotropic hypogonadism
GHD: Growth hormone deficiency
CGHD: Complete growth hormone deficiency
BA: Bone age
CA: Chronological age
SDS: Standard deviation scores
GH: Growth hormone
ACTH: Adrenal corticotropic hormone
TSH: Thyroid-stimulating hormone
LH: Luteinizing hormone
FSH: Follicle stimulating hormone
IGF1: Insulin-like growth factor 1.
